# Rotman Lens-Based Circular Array for Generating Five-mode OAM Radio Beams

**DOI:** 10.1038/srep27815

**Published:** 2016-06-10

**Authors:** Xu-Dong Bai, Xian-Ling Liang, Jian-Ping Li, Kun Wang, Jun-Ping Geng, Rong-Hong Jin

**Affiliations:** 1 Department of Electronics Engineering, Shanghai Jiao Tong University, Shanghai 200240, China

## Abstract

Recently, vortex beam carrying orbital angular momentum (OAM) for radio communications has attracted much attention for its potential of transmitting multiple signals simultaneously at the same frequency, which can be used to increase the channel capacity. However, most of the methods for getting multi-mode OAM radio beams remain up to now mainly at simulation level, since their implementations are of complicated structure and very high cost. This paper provides an effective design of generating five-mode OAM radio beams by using the Rotman lens-fed antenna array. The Rotman lens is a viable beamforming approach instead of electronically scanned arrays for its low cost and the ease of implementation. The lens-fed array employs a two-layer structure for size reduction, and the lens body and the antenna array are segregated by a common ground plane to eliminate spurious radiation and thus improve the performance of the OAM beams. The measured results coincide with the simulated ones, which verified the effectiveness of the proposed design for generating multi-mode OAM beams.

Nowadays, the majority of the human information is transmitted through wireless channels and the fast-increasing mobile devices have led to congestion in the precious radio spectrum resource even after the application of many techniques showing the ability for increasing the spectral efficiency, such as orthogonal frequency-division multiplexing (OFDM)^1^. So recently, orbital angular momentum (OAM) for radio communications has attracted much attention, since it shows potential of transmitting multiple signals simultaneously at the same frequency, which can be used to increase the channel capacity and utilize the radio spectrum with very high efficiency^2^.

An electromagnetic wave radiates energy as well as angular momentum (AM)^3^. The angular momentum is composed of spin angular momentum (SAM) describing the polarization state and orbital angular momentum (OAM) relating to the helical phase structures. Since SAM has been widely used starting around 1935 when it was experimentally demonstrated by Beth^4^, OAM is not fascinating until Allen *et al*. investigated in 1992 that beams with helical wave fronts comprise an azimuthal phase term 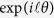 and have an OAM of 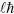 per photon (where 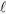 is topological charge, *θ* is the azimuthal angle)^5^. At first, the applications of OAM have mostly been within the optical regime, and by introducing OAM, the capacity of optical communication systems is largely extended^6^,^7^. It was not found until very recently that the photon OAM can be used in the low frequency radio domain and is not restricted to the optical frequency range^8^. From that moment on, many optical methods were introduced to give rise to OAM-carrying radio beams, such as dielectric or FSS spiral phase plate^9^,^10^,^11^, spiral reflector^12^, twisted parabolic reflector^13^,^14^,^15^ and the plane reflectarray^16^; in all these methods, the fixed-mode OAM wave is obtained by a transformation of the plane wave or spherical wave. Another common solution in microwave domain relies mainly on the circular arrays^17^,^18^,^19^,^20^,^21^, and the element is fed with the uniform amplitude but with a successive phase difference. At lower cost, the shunt-fed and series-fed networks are used to feed the circular arrays respectively^17^,^18^,^19^, both of which generate the single-mode OAM waves. To get multi-mode OAM beams, the concept of phased array antenna is introduced to feed the elements with variable phases^20^,^21^; however, this method has remained up to now mainly at simulation level, since the phased array system is usually very complex and of high cost. So instead, a Butler matrix is proposed as feed network of the circular array to generate multi-mode OAM beams^22^, but the design and architecture are also very complicated and composed of many couplers and phase shifters, which restrict it to the initial exploitation stage of the simulation.

In this work, a two-layer Rotman lens-fed antenna array is introduced to generate five-mode OAM radio beams. Through the integrated implementation of the multilayer lens-fed antenna array, a viable solution is obtained for the generation of multi-mode OAM radio beams. The lens-fed array has the advantages of low cost and ease of implementation and integration with available fabrication technologies for integrated circuits. Simulation results and experimental measurements validate the effectiveness of the proposed design for generating multi-mode OAM beams.

## Results

### Design method of the Rotman lens-fed array for multi-mode OAM beams

**Figure 1:**
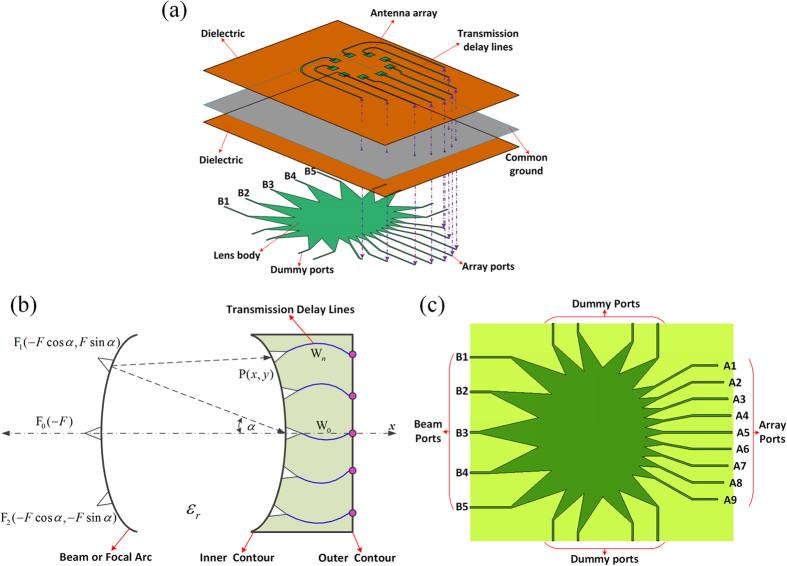
Geometry architecture and design. (**a**) Proposed two-layer Rotman lens-fed antenna array for generating multi-mode OAM beams. (**b**) Schematic diagram and design parameters of the Rotman lens. (**c**) The designed Rotman lens with five beam ports and nine array ports.

The overall configuration of the lens-fed OAM array is shown in Fig. 1(a), which consists of two stacked substrate layers. The circular antenna array is on the top surface of the upper layer, the Rotman lens is positioned on the bottom surface of the lower layer, and a common ground is placed in the middle of the two substrate layers to segregate the direct-radiating array from the lens body and thus eliminate the spurious radiation, which may degrade performance in the OAM modes. The signal transition from the lens to the array through the common ground is implemented in the form of vias. By employing a two-layer structure, the overall dimension of the lens-fed array is greatly reduced, and this method is effective and already widely available^23^,^24^.

The Rotman lens is a succinct and efficient method for the broadband microwave beamforming^24^,^25^,^26^,^27^,^28^. A general layout of the Rotman lens is shown in Fig. 1(b). It has an elaborately designed lens body, formed by the beam arc and the inner contour, and suitable transmission delay lines (*W*_*n*_) from the inner contour to the outer contour to produce a phase difference for the successive outputs by the time delay in the wave transmission. Each input beam port will excite a distinct linear time delay across the output array ports, so getting different phase difference needs only to feed a different beam port. In the conventional design of the Rotman lens, there are a series of design parameters and formulas to ascertain the focal points *F*_1_, *F*_0_, *F*_2_ and ports positions, including the focal angle (*α*), the ratio of off-axis to on-axis focal length (*β*), the number of beams and array ports^25^,^27^,^28^.

So far, the Rotman lens is typically designed for linear electronically scanned arrays^24^,^25^,^26^,^27^. In our work, we adopt the Rotman lens as feed networks for a circular array to generate multi-mode OAM radio beams. To obtain the five-mode OAM radio beams, five beam ports and nine array ports are used to construct the lens, as shown in Fig. 1(c); and eight dummy ports are also added to avoid internal reflection. The Rotman lens is designed to operate at 7.9 GHz in X-band, with a diameter of about 149 mm. The substrate used for the lens is with the dielectric constant of 2.55 and thickness of 0.762 mm, and the detailed parameters of the proposed lens are set as follows: focal angle *α* = 30°, the ratio of off-axis to on-axis focal length *β* = 0.88, and the array ports vertical spacing is 0.5*λ*_0_. During the operation, the array ports are used to feed the antenna array, the dummy ports are terminated with the 50-Ω loads to avoid internal reflection, and each input beam port will produce a constant phase difference for the successive array ports. When port B1 is excited, an average −80° phase difference is generated successively from array port A1 to A9. For port B2 or B3, the phase difference is −40° or 0°, correspondingly. Based on the symmetrical principle, we can easily derive that the phase difference is 40° or 80° when port B4 or B5 is excited.

**Figure 2:**
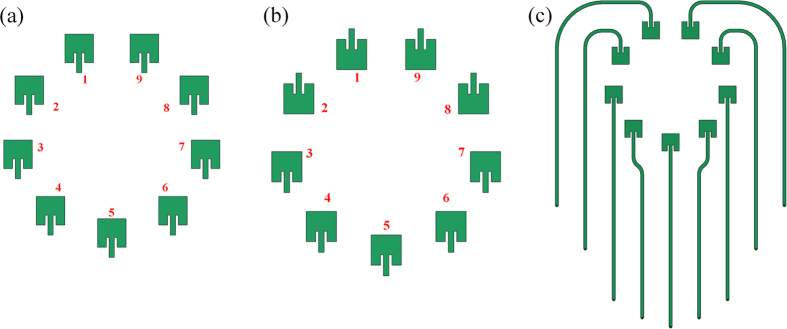
Configuration of the OAM-generating antenna array for D = 2*λ*_*0*_. (**a**) Initial array structure and the elements placement. (**b**) Refined array elements placement. (**c**) Refined array arrangement with transmission delay lines.

For an *N*-element OAM circular array, to get helical wave fronts, the array element should be fed with the uniform amplitude but with a successive phase difference for the successive elements, which is 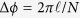
(where 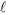 is the topological charge)^8^,^20^. In our work, the circular array consists of nine identical rectangular patches to joint with the Rotman lens. The circular array was also fabricated on a substrate with the dielectric constant of 2.55 and height of 0.762 mm, and the array diameter is *D* = 2*λ*_*0*_. The initial array structure and elements placement is shown in Fig. 2(a). The phase increment of the feed-in signal after a full rotation is 360° for the OAM modes 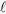 = +1 or −1, and the phase difference for the successive elements is then 40°. For the OAM modes +2 or −2, the phase shift step is 80° from element to element. In the refined design, we make an inversion for elements 1, 2, 8, 9 as shown in Fig. 2(b), in order to reduce the mutual coupling between elements, especially for elements 1 and 2. An additional advantage for this refined array arrangement is that the total size of the lens-fed array can also be reduced, since the length of transmission lines for elements 3–7 is set half-wavelength shorter to compensate the additional 180° phase shift introduced by the reversed array elements. The refined array arrangement with transmission delay lines is shown in Fig. 2(c). For the transmission delay lines between the lens and array, we employ straight line instead of the most common bent lines used in Rotman lens to eliminate the spurious radiation and unwanted phase deviations in the immediate changes of the bent sections, which may have performance deteriorate in the OAM modes.

**Figure 3:**
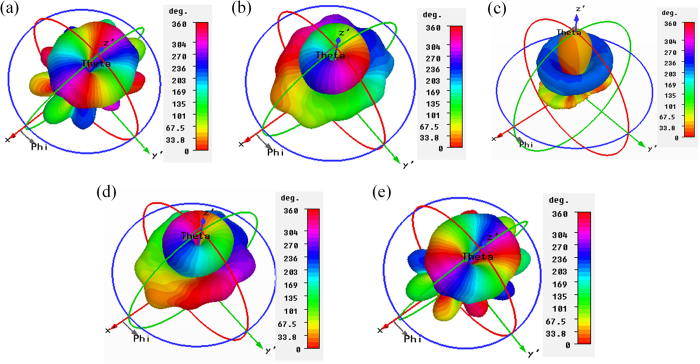
The simulated far-field phase distribution at 7.9 GHz. (**a**) 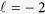. (**b**) 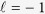. (**c**) 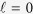. (**d**) 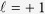. (**e**) 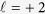.

The simulated far-field phase distribution of the antenna array is shown in Fig. 3, which is simulated with CST Microwave Studio software based on the finite integration technique (FIT) method. It is observed that, the phase distributions of the main lobe are of obvious azimuthal angle dependence for 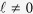, and the changing trends for OAM modes with negative and positive 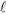 values are in opposite directions.

### Experiment results

**Figure 4:**
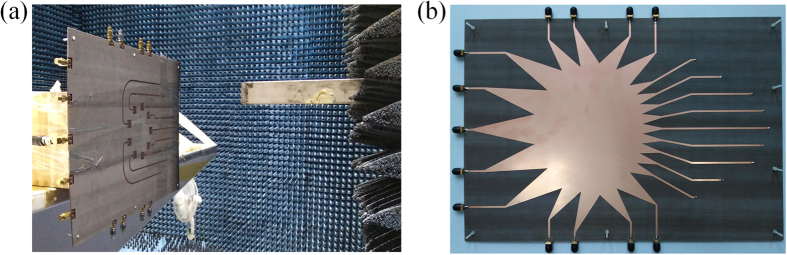
Fabricated prototype of the antenna array. (**a**) Front view and the near-field test scenario in the anechoic chamber. (**b**) Back view.

To verify the proposed design, the prototype lens-fed array was fabricated by using the low-cost commercial printed-circuit-board (PCB) technology, and its measurement is carried out in the anechoic chamber, as shown in Fig. 4.

**Figure 5:**
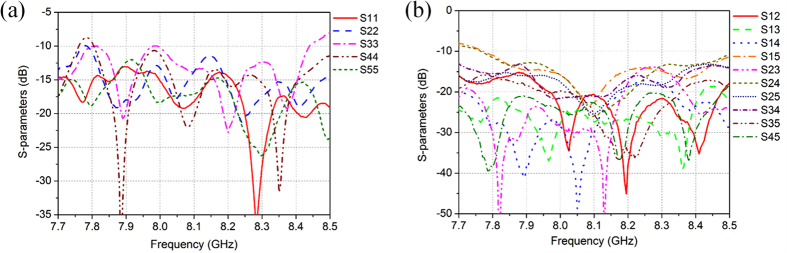
Measured *S*-parameters of the proposed antenna array. (**a**) Return loss. (**b**) Isolation of ports.

Figure 5 shows the measured *S*-parameters, the measured return losses for all five beam ports are higher than 10 dB at 7.8~8.43 GHz, and the ports isolation are higher than 10 dB at 7.78~8.5 GHz.

**Figure 6:**
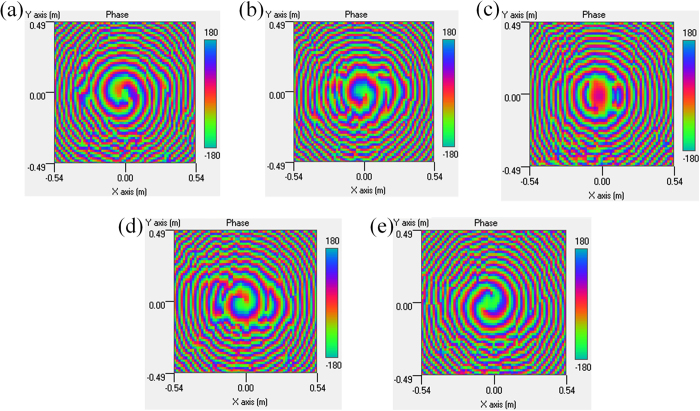
Screenshots of the measured near-field phase distributions at 7.9 GHz. (**a**) 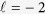. (**b**) 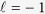. (**c**) 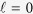. (**d**) 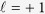. (**e**) 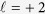.

The near field distribution of the antenna array is measured with a 3D platform in the anechoic chamber, as shown in Fig. 4(a). An open-ended rectangular waveguide is used as the near-field probe, and the measuring plane is 180 *mm* far from the array plane with a scan range of 1080 *mm* × 980 *mm*. Figure 6 gives the screenshots of the measured near-field phase distribution. Note that, for OAM mode 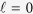, an annular phase fronts is generated, which is the typical characteristic for plane or spherical wave; and for OAM mode 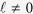, the characteristic vortex phase fronts indicate that the OAM radio beams are created, and the change in color from green to red, blue, and back to green again corresponds to a change in phase of 2*π*. When comparing the positive and negative modes, the phase distribution of 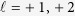 has a clockwise increase, while that of 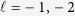 would have an anticlockwise increase. Since the phase distribution of the OAM beam reveals such a distinctive feature, it is easily to determine the OAM mode by analyzing the phase variation. There are also some phase aliasing in Fig. 6, especially for the singular-phase central areas when 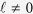, this is mainly due to the limited resolution ratio caused by the larger section of the open-ended waveguide. The dislocations in the measured near-field phase distribution attribute to the limited positional accuracy of the test system.

**Figure 7:**
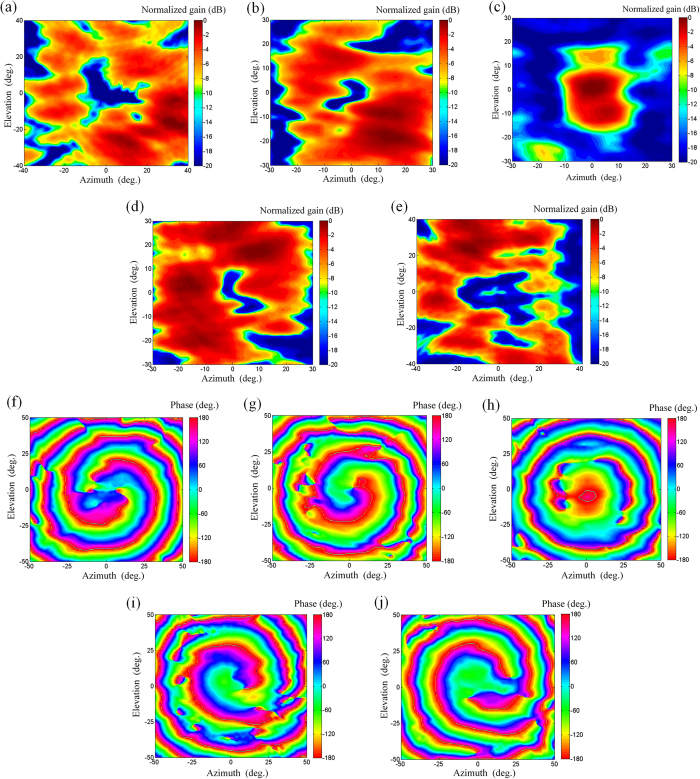
Measured far-field results at 7.9 GHz. Intensity patterns: (**a**) 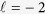. (**b**) 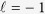. (**c**) 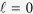. (**d**) 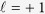. (**e**) 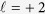. Phase distribution:(**f**) 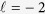. (**g**) 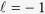. (**h**) 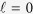. (**i**) 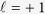. (**j**) 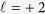.

The far-field radiations of the array are shown in Fig. 7, which are constructed from the near-field measurement by a two-dimensional fast Fourier transform (FFT) algorithm^29^. Figure 7(a–e) gives the intensity patterns, the doughnut-shaped high-intensity profile is clearly seen, and the on-axis null regions of OAM modes 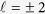 have a clearly larger size than that of 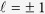. The far-field phase distributions of the array are plotted in Fig. 7(f–j), and the vortex-shaped phase distribution is of obvious azimuthal angle dependence.

**Figure 8:**
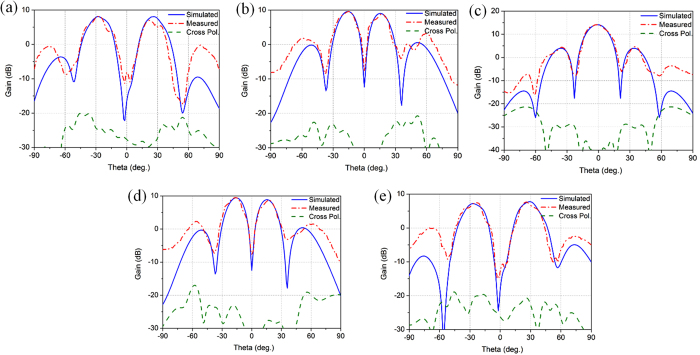
The measured 2D far-field radiation patterns at 7.9 GHz. (**a**) 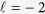. (**b**) 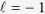. (**c**) 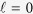. (**d**) 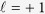. (**e**) 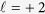.

To further validate the performance of the proposed array, the 2D far-field radiation patterns are measured by a standard horn antenna, and the measured results coincide well with the simulated ones as shown in Fig. 8. Conical patterns are obtained for OAM modes 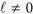, and the peak direction of the main lobe for modes 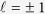 is around 15° from the boresight axis, while the peak direction of modes 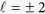 is around 28°. The maximum gain appears on mode 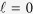, with a measured value of about 14.2 dB; and the array gain decreases as the absolute value 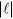 increases. The on-axis null region is over 20 dB lower than the high-intensity doughnut-shaped region for both 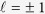 and 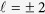. The intensity of the cross-polarization field is much lower than that of the co-polarization field, and the cross polarization levels are lower than −25 dB.

## Discussion

A two-layer Rotman lens-fed antenna array for generating OAM radio waves is presented. Through the integrated implementation of the multilayer lens-fed antenna array, a viable solution is obtained for the generation of multi-mode OAM beams. The lens-fed array has the advantages of low cost and ease of implementation and integration with available fabrication technologies for integrated circuits. All these features make the proposed antenna a promising candidate for radio communications and radar applications.

## Methods

The multi-mode OAM radio beams are generated by using a two-layer Rotman lens-fed antenna array. The lens-fed array is manufactured on a 0.762-mm Arlon AD255 substrate using the PCB technology.

The measurement is carried out with a 3D platform in the anechoic chamber based on the Agilent Vector Network Analyzer 8722ES, and both intensity and phase distributions of the five-mode OAM waves can be measured.

## Additional Information

**How to cite this article**: Bai, X.-D. *et al*. Rotman Lens-Based Circular Array for Generating Five-mode OAM Radio Beams. *Sci. Rep.* **6**, 27815; doi: 10.1038/srep27815 (2016).
